# Strong convergence of gradient projection method for generalized equilibrium problem in a Banach space

**DOI:** 10.1186/s13660-017-1574-x

**Published:** 2017-11-28

**Authors:** Mohammad Farid, Syed Shakaib Irfan, Mohammad Firdosh Khan, Suhel Ahmad Khan

**Affiliations:** 1 Unaizah College of Engineering, Qassim University, Unaizah, 51911 Saudi Arabia; 2 College of Engineering, Qassim University, Buraidah, 51452 Saudi Arabia; 3 Department of Mathematics, BITS-Pilani, Dubai Campus, Dubai, 345055 United Arab Emirates

**Keywords:** generalized equilibrium problem, variational inequality problem, fixed point problem, relatively nonexpansive mappings, iterative scheme

## Abstract

In this paper, we propose and analyze a hybrid iterative method for finding a common element of the set of solutions of a generalized equilibrium problem, the set of solutions of a variational inequality problem, and the set of fixed points of a relatively nonexpansive mapping in a real Banach space. Further, we prove the strong convergence of the sequences generated by the iterative scheme. Finally, we derive some consequences from our main result. Our work is an improvement and extension of some previously known results recently obtained by many authors.

## Introduction

Let *X* be a real Banach space with its dual space $X^{*}$, let $\langle \cdot,\cdot\rangle$ be the duality pairing between *X* and $X^{*}$, and let $\|\cdot\|$ denote the norm of *X* and $X^{*}$. Let *K* be a nonempty closed convex subset of *X*, and let $2^{X}\neq\emptyset$ be the set of all subsets of *X*.

Let $G, \xi:K\times K\to\mathbf{R}$ be bifunctions. The generalized equilibrium problem (GEP) is finding $x \in K$ such that

1.1 $$ G(x,y)+\xi(y,x)-\xi(x,x)\geq0,\quad \forall y \in K. $$

We denote the solution set of GEP (1.1) by Sol(GEP(1.1)). Problem (1.1) includes fixed point problems, optimization problems, variational inequality problems, Nash equilibrium problems, *etc*. as particular cases. In recent past, many iterative methods have been proposed to solve GEP (1.1); see, for example, [1–4].

For $\xi=0$, GEP (1.1) reduces to the following equilibrium problem (EP): Find $x\in K$ such that

1.2 $$ G(x,y)\geq0 \quad\text{for all } y\in K. $$

Problem (1.2) was introduced and studied by Blum and Oettli [5].

The variational inequality problem (VIP) is to find $x\in K$ such that

1.3 $$ \langle Sx, y-x \rangle\geq0 \quad\text{for all } y\in K, $$

where $S:K\to X^{*}$ is a nonlinear mapping. We denote the solution set of VIP (1.3) by Sol(VIP(1.3)).

A mapping $S:K\to X^{*}$ is said to be

(i) monotone if $\langle x-y, Sx-Sy \rangle\geq0$ for all $x,y\in K$;
(ii) *γ*-inverse strongly monotone if there exists a positive real number *γ* such that $\langle x-y, Sx-Sy \rangle\geq\gamma\|Sx-Sy\|^{2}$ for all $x,y\in K$;
(iii) Lipschitz continuous if there exists a constant $L>0$ such that $\|Sx-Sy\|\leq L\|x-y\|$.

If *S* is *γ*-inverse strongly monotone, then it is Lipschitz continuous with constant $\frac{1}{\gamma}$, that is, $\|Sx-Sy\|\leq\frac{1}{\gamma}\|x-y\|$ for all $x,y\in K$.

The *fixed point problem* (FPP):

1.4 $$ \text{Find } x\in K \quad\text{such that } x\in\operatorname{Fix}(T), $$

where $T:K\to K$ is a nonlinear mapping, and $\operatorname{Fix}(T)$ is the fixed point set.

In 2009, Takahashi and Zembayashi [1] studied weak and strong convergence theorems for finding a common solution of EP (1.2) and FPP (1.4) of a relatively nonexpansive mapping in a real Banach space. Later on, Petrot *et al*. [2] extended the work [1] by using the hybrid projection method, which plays an important role for establishing strong convergence results.

Nadezhkina *et al*. [6] proposed a convex combination of a nonexpansive mapping and the extragradient method and considered the iterative scheme by the hybrid method. They proved the strong convergence theorem in a Hilbert space.

Very recently, in 2015, Nakajo *et al*. [7] proposed a composition and convex combination of a relatively nonexpansive mapping and the gradient method. Further, they proved the strong convergence to a common element of solutions of the variational inequality problem and fixed point problem by using the hybrid method.

Motivated and inspired by the recent work of Takahashi and Zembayashi [1], Petrot *et al*. [2], Nadezhkina *et al*. [6], and Nakajo *et al*. [7], we propose an iterative scheme to find the common solution of GEP (1.1), VIP (1.3), and FPP (1.4) for a relatively nonexpansive mapping in a real Banach space. Further, by using the hybrid projection we prove the strong convergence of the sequences generated by the iterative algorithm, which improves and extends the corresponding results of [3, 4, 8–10].

## Preliminaries

Now, we use the following results and definitions to prove our main result.

The normalized duality mapping is defined as

$$J(u)=\bigl\{ v\in X^{*}:\langle u,v \rangle=\|u\|^{2}=\|v \|^{2}\bigr\} $$

for every $u\in X$, where $J:X\to2^{X^{*}}$.

The mapping $\rho_{X}:[0, \infty)\to[0, \infty) $ defined by

$$\rho_{X}(s)=\sup \biggl\{ \frac{\|u+v\|+\|u-v\|}{2}-1:\|u\|=1, \|v\|=s \biggr\} $$

is called the modulus of smoothness of *X*. The space *X* is said to be smooth if $\rho_{X}(s)> 0$ for all $s > 0$, and *X* is called uniformly smooth if $\frac{\rho_{X}(s)}{s}\to0$ as $s\to0$. A Banach space *X* is said to be *q*-uniformly smooth if there exists a fixed constant $c>0$ such that $\rho_{X}(s)\leq c s^{q}$. It is well known that if *X* is *q*-uniformly smooth, then $q\leq2$, and *X* is uniformly smooth. Note that if *X* is uniformly smooth, then *J* is uniformly continuous on bounded subsets of *X*.

The modulus of convexity of *X* is the function $\delta_{X}:(0, 2]\to [0, 1]$ defined by

$$\delta_{X}(t)=\inf \biggl\{ 1-\frac{1}{2}\|x+y\|: \|x\| = \|y \|=1, \|x-y\| =t \biggr\} $$

for $t\in(0, 2]$. A Banach space *X* is said to be uniformly convex if $\delta_{X}(t)>0$ for all $t\in(0, 2]$. Let $p>1$. The space *X* is said to be *p*-uniformly convex if there exists a constant $c>0$ such that $\delta_{X}(t)\geq ct^{p}$ for all $t\in(0, 2]$. Note that every *p*-uniformly convex space is uniformly convex (for more details, see [11]).

Let *X* be a smooth, strictly convex, and reflexive Banach space.

Following Takahashi and Zembayashi [1], a point $x_{0}\in K$ is said to be an asymptotic fixed point of *T* if *K* contains a sequence $\{x_{n}\}$ that converges weakly to $x_{0}$ such that $\lim_{n\to\infty}\|x_{n}-Tx_{n}\|=0$. The set of asymptotic fixed points of *T* is denoted by $\widehat{\operatorname{Fix}}(T)$. A mapping *T* from *K* into itself is said to be relatively nonexpansive if $\operatorname{Fix}(T)\neq\emptyset$, $\widehat{\operatorname {Fix}}(T)=\operatorname{Fix}(T)$, and $\phi(x_{0},Tx)\leq\phi (x_{0},x)$ for all $x\in K$ and $x_{0}\in\operatorname{Fix}(T) $, where $\phi:X\times X\to\mathbf{R}_{+} $ is the Lyapunov functional defined by

2.1 $$ \phi(u,v)=\|u\|^{2}-2\langle u,Jv\rangle+\|v\|^{2}\quad \text{for } u,v\in X. $$

The generalized projection $\Pi_{K}:X\to K$ is defined as

$$\Pi_{K}(u)=\inf_{x\in K} \phi(u,x) \quad\text{for } u\in X, $$

where $\phi(u,x)$ is defined by (2.1) (for more details, see [12]).

### Lemma 2.1

([12, 13])

*Let* *X* *be a smooth*, *strictly convex*, *and reflexive Banach space*, *and let* $K\neq\emptyset$ *be a closed convex subset of* *X*. *Then*, *the following hold*:

(i) $\phi(x,\Pi_{K}u)+\phi(\Pi_{K}u,u)\leq\phi(x,u)$ *for all* $x\in K$, $u\in X$.
(ii) *For* $u\in X$ *and* $x\in K$, *we have*
  $$x=\Pi_{K}(u)\quad\Leftrightarrow\quad\langle x-y,Ju-Jx\rangle\geq0 \quad\textit{for all } y\in K. $$

### Remark 2.1

([1])

(i) Using (2.1), we get
  $$\bigl(\|u\|-\|v\|\bigr)^{2}\leq\phi(u,v)\leq\bigl(\|u\|+\|v\|\bigr)^{2}\quad \text{for all } u,v\in X. $$
(ii) If $X=H$ is a real Hilbert space, then $\phi(u,v)=(\|u\| -\|v\|)^{2}$, and $\Pi_{K}=P_{K}$, the metric projection of *H* onto *K*.
(iii) If *X* is a smooth, strictly convex, and reflexive Banach space, then $\phi(u,v)=0$ for $u,v\in X $ if and only if $u=v$.

### Lemma 2.2

([11])

*Let* *X* *be a smooth Banach space*. *Then*, *the following are equivalent*:

(i) *X* *is* 2-*uniformly convex*.
(ii) *There exists a constant* $c_{1}>0$ *such that* $\|u+v\| ^{2}\geq\|u\|^{2}+2\langle v,Ju \rangle+c_{1}\|v\|^{2}$ *for all* $u, v\in X$.

### Lemma 2.3

([11])

*Let* *X* *be a* 2-*uniformly convex and smooth Banach space*. *Then* $\phi(u,v)\geq c_{1}\|u-v\|^{2}$ *for all* $u, v\in X$, *where* $c_{1}$ *is the constant in Lemma* 2.2.

### Lemma 2.4

([11])

*Let* *X* *be a* 2-*uniformly convex Banach space*. *Then*, *for all* $u, v\in X$, *we have*

$$\|u-v\|\leq\frac{2}{c_{1}^{2}}\|Ju-Jv\|, $$

*where* *J* *is the normalized duality mapping of* *X*, *and* $0< c_{1}\leq1$.

### Lemma 2.5

([10])

*Let* $K\neq\emptyset$ *be a closed convex subset of a smooth*, *strictly convex*, *and reflexive Banach space* *X*, *and let* $T:K\to K$ *be a relatively nonexpansive mapping*. *Then*, $\operatorname{Fix}(T)$ *is closed and convex*.

### Lemma 2.6

([13])

*Let* *X* *be a smooth and uniformly convex Banach space*, *and let* $\{u_{n}\}$ *and* $\{v_{n}\}$ *be sequences in* *X* *such that either* $\{u_{n}\}$ *or* $\{v_{n}\}$ *is bounded*. *If* $\lim_{n\to\infty}\phi(u_{n},v_{n})=0$, *then* $\lim_{n\to\infty}\|u_{n}-v_{n}\|=0$.

### Lemma 2.7

([14])

*Let* *K* *be a nonempty closed convex subset of a Banach space* *X*, *and let* *S* *be a monotone and hemicontinuous operator of* *K* *into* $X^{*}$. *Define the mapping* $M\subset X\times X^{*}$ *as*

$$M(z) =\left \{ \textstyle\begin{array}{l@{\quad}l} S(z)+N_{K}(z) &\textit{if } z \in K , \\ \emptyset &\textit{if } z \notin K, \end{array}\displaystyle \right . $$

*where* $N_{K}(z):=\{u\in X^{*}:\langle z-x,u\rangle\geq0, \forall x\in K\}$ *is the normal cone to* *K* *at* $z \in K$. *Then*, *M* *is maximal monotone*, *and* $M^{-1}(0)=\textit{Sol}(\textit{VIP}(\text{1.3}))$.

### Lemma 2.8

([11, 15])

*Let* *X* *be a uniformly convex Banach space*, *and let* $r>0$. *Then there exists a strictly increasing*, *continuous*, *and convex function* $g:[0,2r]\to\mathbf{R}$ *such that* ${g(0)=0}$ *and*

$$\big\| \alpha x+(1-\alpha)y\big\| ^{2}\leq\alpha\|x\|^{2}+(1-\alpha) \|y\| ^{2}-\alpha(1-\alpha)g\bigl(\|x-y\|\bigr) $$

*for all* $x,y\in B_{r}$ *and* $\alpha\in[0,1]$, *where* $B_{r}=\{u\in X:\|u\| \leq r\}$.

### Lemma 2.9

([13])

*Let* *X* *be a smooth and uniformly convex Banach space*, *and let* $r>0$. *Then there exists a strictly increasing*, *continuous*, *and convex function* $g:[0,2r]\to\mathbf{R}$ *such that* $g(0)=0$ *and*

$$g\bigl(\|x-y\|\bigr)\leq\phi(x,y) \quad\textit{for all } x,y\in B_{r}. $$

The function $F:X\times X^{*}\to\mathbf{R} $ defined by

$$F\bigl(u,u^{*}\bigr)=\|u\|^{2}-2\bigl\langle u,u^{*} \bigr\rangle +\big\| u^{*}\big\| ^{2} \quad\mbox{for } u\in X \text{ and } u^{*}\in X^{*} $$

was studied by Alber [12], that is, $F(u,u^{*})=\phi (u,J^{-1}u^{*})$ for $u\in X$ and $u^{*}\in X^{*}$.

### Lemma 2.10

([12])

*Let* *X* *be a reflexive strictly convex and smooth Banach space with its dual* $X^{*}$. *Then*

$$G\bigl(u,u^{*}\bigr)+2\bigl\langle J^{-1}u^{*}-u,v^{*} \bigr\rangle \leq G\bigl(u,u^{*}+v^{*}\bigr) \quad\textit{for all } u \in X \textit{ and } u^{*},v^{*}\in X^{*}. $$

### Assumption 2.1

Let *G* and *ξ* satisfy the following conditions:

(i) $G(x,x)=0$ for $x \in K$.
(ii) *G* is monotone, that is, $G(x,y)+G(y,x)\leq0$ for $x,y \in K$.
(iii) For each $y\in K$, $x\rightarrow G(x,y)$ is weakly upper semicontinuous.
(iv) For each $x\in K$, $y\rightarrow G(x,y)$ is convex and lower semicontinuous.
(v) $\xi(\cdot,\cdot)$ is weakly continuous, and $\xi(\cdot ,y)$ is convex.
(vi) *ξ* is skew-symmetric, that is,
  $$\xi(x,x)-\xi(x,y)+\xi(y,y)-\xi(y,x)\geq0 \quad\mbox{for all } x,y \in K. $$

### Theorem 2.1

*Let* *K* *be a nonempty closed and convex subset of a smooth*, *strictly convex*, *and reflexive Banach space* *X*. *Let* $G,\xi:K\times K\to\mathbf{R}$ *be nonlinear mappings satisfying Assumption* 2.1. *For* $t>0$ *and* $u\in X$, *define the mapping* $\Upsilon _{t}:X\to K$ *as follows*:

$$\Upsilon_{t}(u)=\biggl\{ z\in K:G(z,y)+\xi(y,z)-\xi(z,z)+ \frac{1}{t}\langle y-z, Jz-Ju\rangle\geq0, \forall y\in K\biggr\} . $$

*Then*, *the following conclusions hold*:

(i) $\Upsilon_{t}$ *is single*-*valued*;
(ii) $\Upsilon_{t}$ *is firmly nonexpansive mapping*, *that is*, *for all* $u_{1}, u_{2}\in X$,
  $$\langle\Upsilon_{t}u_{1}-\Upsilon_{t}u_{2},J \Upsilon_{t}u_{1}-J\Upsilon _{t}u_{2} \rangle\leq\langle\Upsilon_{t}u_{1}-\Upsilon _{t}u_{2},Ju_{1}-Ju_{2} \rangle; $$
(iii) $\operatorname{Fix}(\Upsilon_{t})=\textit{Sol}(\textit{GEP}(\text{1.1}))$;
(iv) *Sol*(*GEP*(1.1)) *is closed and convex*.

### Proof

(i) We claim that $\Upsilon_{t}$ is single-valued. Indeed, for $x\in K$ and $t>0$, let $z_{1}, z_{2}\in\Upsilon_{t}(x)$. Then

2.2 $$ G(z_{1},y)+\xi(y,z_{1})-\xi(z_{1},z_{1})+ \frac{1}{t}\langle y-z_{1},Jz_{1}-Jx \rangle\geq0 \quad\mbox{for all } y\in K $$

and

2.3 $$ G(z_{2},y)+\xi(y,z_{2})-\xi(z_{2},z_{2})+ \frac{1}{t}\langle y-z_{2},Jz_{2}-Jx \rangle\geq0 \quad\mbox{for all } y\in K. $$

Letting $y=z_{2}$ in (2.2) and $y=z_{1}$ in (2.3) and then adding, we have

$$G(z_{1},z_{2})+G(z_{2},z_{1})+ \xi(z_{2},z_{1})-\xi(z_{1},z_{1})+\xi (z_{1},z_{2})-\xi(z_{2},z_{2})+ \frac{1}{t}\langle z_{2}-z_{1},Jz_{1}-Jz_{2} \rangle\geq0. $$

Since *G* is monotone, *ξ* is skew symmetric, and since $t>0$, we have

$$\langle z_{2}-z_{1},Jz_{1}-Jz_{2} \rangle\geq0. $$

Using the strict convexity of *X*, we get $z_{1}=z_{2} $. Thus, $\Upsilon_{t}$ is single-valued.

(ii) For any $u_{1}, u_{2}\in X$, let $x_{1}=\Upsilon_{t}u_{1}$ and $x_{2}=\Upsilon_{t}u_{2}$. Then

2.4 $$ G(x_{1},y)+\xi(y,x_{1})-\xi(x_{1},x_{1})+ \frac{1}{t}\langle y-x_{1},Jx_{1}-Ju_{1} \rangle\geq0 \quad\mbox{for all } y\in K, $$

and

2.5 $$ G(x_{2},y)+\xi(y,x_{2})-\xi(x_{2},x_{2})+ \frac{1}{t}\langle y-x_{2},Jx_{2}-Ju_{2} \rangle\geq0 \quad\mbox{for all } y\in K. $$

By putting $y=x_{2}$ in (2.4) and $y=x_{1}$ in (2.5) and taking their sum, we have

$$\begin{gathered} G(x_{1},x_{2})+G(x_{2},x_{1})+ \xi(x_{2},x_{1})-\xi(x_{1},x_{1})+\xi (x_{1},x_{2})-\xi(x_{2},x_{2}) \\\quad{}+\frac{1}{t}\langle x_{2}-x_{1},Jx_{1}-Jx_{2}-Ju_{1}+Ju_{2} \rangle\geq0.\end{gathered} $$

Using the monotonicity of *G* and properties of *ξ*, we have

$$\frac{1}{t}\langle x_{2}-x_{1},Jx_{1}-Jx_{2}-Ju_{1}-Ju_{2} \rangle\geq0. $$

Hence, we have

$$\langle x_{2}-x_{1},Jx_{1}-Jx_{2} \rangle+\langle x_{2}-x_{1},Ju_{2}-Ju_{1} \rangle\geq0 $$

or

$$\langle x_{1}-x_{2},Jx_{1}-Jx_{2} \rangle\leq\langle x_{1}-x_{2},Ju_{1}-Ju_{2} \rangle, $$

that is,

2.6 $$ \langle\Upsilon_{t}u_{1}-\Upsilon_{t}u_{2},J \Upsilon_{t}u_{1}-J\Upsilon _{t}u_{2} \rangle\leq\langle\Upsilon_{t}u_{1}-\Upsilon _{t}u_{2},Ju_{1}-Ju_{2} \rangle. $$

Thus, $\Upsilon_{t}$ is a firmly nonexpansive mapping.

(iii) Let $x\in\operatorname{Fix}(\Upsilon_{t})$. Then

$$G(x,y)+\xi(y,x)-\xi(x,x)+\frac{1}{t}\langle y-x,Jx-Jx \rangle\geq0 \quad\mbox{for all } y\in K, $$

and so

$$G(x,y)+\xi(y,x)-\xi(x,x)\geq0 \quad\mbox{for all } y\in K . $$

Thus, $x\in\text{Sol(GEP(1.1))}$.

Let $x\in\text{Sol(GEP(1.1))}$. Then

$$G(x,y)+\xi(y,x)-\xi(x,x)\geq0 \quad\mbox{for all } y\in K, $$

and so

$$G(x,y)+\xi(y,x)-\xi(x,x)+\frac{1}{t}\langle y-x,Jx-Jx \rangle\geq0\quad \text{for all } y\in K . $$

Hence, $x\in\operatorname{Fix}(\Upsilon_{t})$. Thus, $\operatorname {Fix}(\Upsilon_{t})=\text{Sol(GEP(1.1))}$.

(iv) First, we show that $\Upsilon_{t}$ is a relatively nonexpansive mapping.

Using the definition of *ξ*, for any $u_{1}, u_{2}\in X$, we have

$$\begin{gathered} \phi(\Upsilon_{t}u_{1},\Upsilon_{t}u_{2})+ \phi(\Upsilon _{t}u_{1},\Upsilon_{t}u_{2})\\\quad=2 \|\Upsilon_{t}u_{1}\|^{2}-2\langle \Upsilon_{t}u_{1},J\Upsilon_{t}u_{2} \rangle -2\langle\Upsilon_{t}u_{2},J\Upsilon_{t}u_{1} \rangle+2\|\Upsilon _{t}u_{2}\|^{2} \\ \quad=2\langle\Upsilon_{t}u_{1},J\Upsilon_{t}u_{1}-J \Upsilon _{t}u_{2}\rangle +2\langle\Upsilon_{t}u_{2},J\Upsilon_{t}u_{2}-J \Upsilon _{t}u_{1}\rangle \\ \quad=2\langle\Upsilon_{t}u_{1}-\Upsilon_{t}u_{2},J \Upsilon _{t}u_{1}-J\Upsilon_{t}u_{2} \rangle\end{gathered} $$

and

$$\begin{gathered} \phi(\Upsilon_{t}u_{1},u_{2})+\phi( \Upsilon_{t}u_{2},u_{1})-\phi ( \Upsilon_{t}u_{1},u_{1})-\phi( \Upsilon_{t}u_{2},u_{2}) \\ \quad=\|\Upsilon_{t}u_{1}\|^{2}-2\langle \Upsilon_{t}u_{1},Ju_{2}\rangle+\| u_{2} \|^{2}+\|\Upsilon_{t}u_{2}\|^{2}+ \|u_{1}\|^{2} \\ \qquad{}-2\langle\Upsilon_{t}u_{2},Ju_{1} \rangle-\| \Upsilon_{t}u_{2}\| ^{2}+2\langle \Upsilon_{t}u_{2},Ju_{2} \rangle-\|u_{2} \|^{2} \\ \qquad{}-\|\Upsilon_{t}u_{1}\|^{2}+2\langle \Upsilon_{t}u_{1},Ju_{1} \rangle - \|u_{1}\|^{2} \\ \quad=2\langle\Upsilon_{t}u_{1},Ju_{1}-Ju_{2} \rangle-2\langle\Upsilon _{t}u_{2},Ju_{1}-Ju_{2} \rangle \\ \quad=2\langle\Upsilon_{t}u_{1}-\Upsilon_{t}u_{2},Ju_{1}-Ju_{2} \rangle.\end{gathered} $$

Since $\Upsilon_{t}$ is firmly nonexpansive, from the above two equalities we have

$$\phi(\Upsilon_{t}u_{1},\Upsilon_{t}u_{2})+ \phi(\Upsilon _{t}u_{2},\Upsilon_{t}u_{1}) \leq\phi(\Upsilon_{t}u_{1},u_{2})+\phi ( \Upsilon_{t}u_{2},u_{1})-\phi( \Upsilon_{t}u_{1},u_{1})-\phi(\Upsilon _{t}u_{2},u_{2}). $$

Thus,

$$\phi(\Upsilon_{t}u_{1},\Upsilon_{t}u_{2})+ \phi(\Upsilon _{t}u_{2},\Upsilon_{t}u_{1}) \leq\phi(\Upsilon_{t}u_{1},u_{2})+\phi ( \Upsilon_{t}u_{2},u_{1}). $$

Taking $u_{2}=u\in\operatorname{Fix}(\Upsilon_{t}) $, we have

$$\phi(u,\Upsilon_{t}u_{1})\leq\phi(u,u_{1}). $$

Further, we prove that $\widehat{\operatorname{Fix}}(\Upsilon _{t})=\text{Sol(GEP(1.1))}$.

Let $x\in\widehat{\operatorname{Fix}}(\Upsilon_{t})$. Then there exists a sequence $\{u_{n}\}\subset X$ such that $u_{n}\rightharpoonup x$ and $\lim_{n\to\infty}\|u_{n}-\Upsilon_{t}u_{n}\|=0$. Thus, $\Upsilon_{t}u_{n}\rightharpoonup x$. Hence, we get $x\in K$.

Since *J* is uniformly continuous on bounded sets, we have

$$\lim_{n\to\infty}\frac{\|Ju_{n}-J\Upsilon_{t}u_{n}\|}{t} = 0,\quad t>0. $$

From the definition of $\Upsilon_{t}$, for any $y\in K$, we have

$$G(\Upsilon_{t}u_{n},y)+\xi(y,\Upsilon_{t}u_{n})- \xi(\Upsilon _{t}u_{n},\Upsilon_{t}u_{n})+ \frac{1}{t}\langle y-\Upsilon _{t}u_{n},J \Upsilon_{t}u_{n}-Ju_{n} \rangle\geq0. $$

Let $y_{p}= (1-p)x+py$ for $p\in(0,1]$. Since $y\in K$ and $x\in K$, we have $y_{p}\in K$, and thus

$$G(\Upsilon_{t}u_{n},y_{p})+\xi(y_{p}, \Upsilon_{t}u_{n})-\xi(\Upsilon _{t}u_{n}, \Upsilon_{t}u_{n})+\frac{1}{t}\langle y_{p}- \Upsilon _{t}u_{n},J\Upsilon_{t}u_{n}-Ju_{n} \rangle\geq0. $$

Since *ξ* is weakly continuous and *G* is weakly lower semicontinuous in the second argument, letting ${n\to\infty}$, we get

$$\begin{gathered} G(x,y_{p})+\xi(y_{p},x)-\xi(x,x) \geq 0, \\ \xi(y_{p},x)-\xi(x,x)\geq G(y_{p},x).\end{gathered} $$

For $p > 0$, we have

$$\begin{aligned} 0&=G(y_{p}, y_{p}) \\ &\leq p G(y_{p}, y)+(1-p)G(y_{p}, x) \\ &\leq p G(y_{p}, y)+(1-p)\bigl[\xi(y_{p},x)-\xi(x,x)\bigr] \\ &\leq p G(y_{p}, y)+(1-p)p\bigl[\xi(y,x)-\xi(x,x)\bigr] \\ &\leq p \bigl[G(y_{p}, y)+(1-p) \bigl(\xi(y,x)-\xi(x,x)\bigr)\bigr].\end{aligned} $$

Dividing by $p>0$ and letting $p\to0_{+}$, we have

$$G(x,y)+\xi(y,x)-\xi(x,x)\geq0. $$

This implies that $x\in\text{Sol(GEP(1.1))}$, and hence $\operatorname{Fix}(\Upsilon_{t})= \text{Sol(GEP(1.1))}= \widehat {\operatorname{Fix}}(\Upsilon_{t})$. Thus, $\Upsilon_{t}$ be a relatively nonexpansive mapping. By Lemma 2.5, $\text{Sol(GEP(1.1))}=\operatorname{Fix } (\Upsilon_{t})$ is closed and convex. □

Next, we have the following lemma whose proof is on the similar lines of the proof of Lemma 2.9 [1] and hence omitted.

### Lemma 2.11

*Let* *X*, *K*, *G*, *ξ*, $\Upsilon_{t}$ *be same as in Theorem* 2.1, *and let* $t>0$. *Then*, *for* $x\in X$ *and* $u\in \operatorname{Fix}(\Upsilon_{t})$, *we have*

$$\phi(u, \Upsilon_{t}x )+\phi(\Upsilon_{t}x, x)\leq \phi(u, x). $$

## Main result

Now, we prove the following convergence theorem.

### Theorem 3.1

*Let* *X* *be a* 2-*uniformly convex and uniformly smooth Banach space*, *and let* *K* *be a nonempty closed and convex subset of* *X*. *Let* $S:K\to X^{*}$ *be a* *γ*-*inverse strongly monotone mapping with constant* $\gamma\in(0,1)$, *let* $G, \xi:K\times K\to\mathbf{R}$ *be nonlinear mappings satisfying Assumption* 2.1, *and let* $T:K\to K$ *be a relatively nonexpansive mapping such that* $\Gamma :=\textit{Sol}(\textit{GEP}(\text{1.1}))\cap\textit{Sol}(\textit{VIP}(\text{1.3}))\cap\operatorname {Fix}(T) \neq\emptyset$. *Let the iterative sequence* $\{x_{n}\}$ *be generated as follows*:

$$\begin{aligned} \begin{aligned} &x_{0}=x\in K , \\ &z_{n}=\prod_{K}J^{-1}(Jx_{n}- \lambda_{n}Sx_{n}), \\ &y_{n}=J^{-1}\bigl(\theta_{n}Jx_{n}+(1- \theta_{n})JTz_{n}\bigr), \\ &u_{n}\in K \end{aligned} \end{aligned}$$

*such that*

$$\begin{aligned}& G(u_{n},y)+\xi(y,u_{n})-\xi(u_{n},u_{n})+ \frac{1}{t_{n}}\langle y-u_{n},Ju_{n}-Jy_{n} \rangle\geq0,\quad \forall y\in K, \\& P_{n}=\bigl\{ z\in K:\phi(z,u_{n})\leq \phi(z,x_{n})-(1-\theta_{n})\phi (z_{n},x_{n}) -2(1-\theta_{n})\lambda_{n}\langle z_{n}-z, Sx_{n}-Sz_{n}\rangle\bigr\} , \\& Q_{n}=\bigl\{ z\in K:\langle x_{n}-z,Jx-Jx_{n} \rangle\geq0\bigr\} , \\& x_{n+1}=\prod_{P_{n}\cap Q_{n}}x, \quad\forall n\in N\cup \{0\}, \end{aligned}$$

*where* *J* *is the normalized duality mapping on* *X*, $t_{n}\in(0,\infty )$, *and* $\{\lambda_{n}\}$ *and* $\{\theta_{n}\}$ *are the sequences in* $(0, \infty)$ *and* (0,1) *satisfying the following*:

(i) $0<\liminf_{n\to\infty}\lambda_{n}\leq\limsup_{n\to\infty }\lambda_{n}<\frac{c_{1}^{2}\gamma}{2}$, *where* $c_{1}$ *is the constant in Lemma* 2.2;
(ii) $0<\liminf_{n\to\infty}\theta_{n}\leq\limsup_{n\to\infty }\theta_{n}<1 $.

*Then*, $\{x_{n}\}$ *converges strongly to* $\prod_{\Gamma} x$, *where* $\prod_{\Gamma}x$ *is the generalized projection of* *X* *onto *Γ.

### Proof

Since *T* is a relatively nonexpansive mapping from *K* into itself, it follows from Lemma 2.5 and Theorem 2.1(iv) that Γ is closed and convex. First, we show that $P_{n}\cap Q_{n}$ is closed and convex for all $n\in N\cup\{0\}$. By the definition of $Q_{n}$ it is closed and convex. Further, by the definition of *ϕ* we observe that $P_{n}$ is closed and

$$\begin{gathered} \phi(z,u_{n})\leq\phi(z,x_{n})-(1-\theta_{n}) \phi (y_{n},x_{n})-2(1-\theta_{n}) \lambda_{n}\langle y_{n}-z, Sx_{n}-Sy_{n} \rangle, \\\|z\|^{2}-2\langle z, Jx_{n} \rangle+\|x_{n} \|^{2}-(1-\theta_{n})\phi (y_{n},x_{n})-2(1- \theta_{n})\lambda_{n}\langle y_{n}-z, Sx_{n}-Sy_{n}\rangle \\\quad{}- \|z\|^{2}+2\langle z, Ju_{n} \rangle-\|u_{n} \|^{2}\geq0, \\2\langle z, Ju_{n}-Jx_{n} \rangle-2(1- \theta_{n})\lambda_{n}\langle y_{n}-z, Sx_{n}-Sy_{n}\rangle+\|x_{n}\|^{2}- \|u_{n}\|^{2}-(1-\theta _{n})\phi(y_{n},x_{n}) \geq0,\end{gathered} $$

and hence $P_{n}$ is closed and convex for all $n \in N\cup\{0\}$. Thus, $P_{n}\cap Q_{n}$ is closed and convex for all $n \in N\cup\{0\}$.

Next, we show that $\Gamma\subset P_{n}\cap Q_{n}$ and $\{x_{n}\}$ is well defined.

Let $x^{*}\in\Gamma$. Then $x^{*}\in\text{Sol(VIP(1.3))}$, that is, $\langle z_{n}-x^{*},Sz_{n} \rangle\geq\langle z_{n}-x^{*},Sx^{*} \rangle\geq0$.

Since $x^{*}\in\Gamma$, using Lemma 2.1, we have

$$\bigl\langle z_{n}-x^{*},Jx_{n}-Jz_{n}- \lambda_{n}Sx_{n} \bigr\rangle \geq0. $$

Thus,

$$\begin{aligned} \bigl\langle z_{n}-x^{*},Jx_{n}-Jz_{n} \bigr\rangle &\geq\lambda_{n}\bigl\langle z_{n}-x^{*},Sx_{n} \bigr\rangle \\ &=\lambda_{n}\bigl\langle z_{n}-x^{*},Sx_{n}-Sz_{n} \bigr\rangle +\lambda _{n}\bigl\langle z_{n}-x^{*},Sz_{n} \bigr\rangle \\ &\geq\lambda_{n}\bigl\langle z_{n}-x^{*},Sx_{n}-Sz_{n} \bigr\rangle \end{aligned} $$

for each $n\in N\cup\{0\}$, which implies

3.1 $$\begin{aligned}& -2\bigl\langle z_{n}-x^{*},Jx_{n}-Jz_{n} \bigr\rangle \leq-2\lambda_{n}\bigl\langle z_{n}-x^{*},Sx_{n}-Sz_{n} \bigr\rangle , \\& 2\bigl\langle x^{*},Jx_{n}-Jz_{n} \bigr\rangle -2 \langle z_{n},Jx_{n}-Jz_{n} \rangle\leq-2 \lambda_{n}\bigl\langle z_{n}-x^{*},Sx_{n}-Sz_{n} \bigr\rangle , \\& 2\bigl\langle x^{*},Jx_{n}-Jz_{n} \bigr\rangle +2 \langle z_{n}, Jz_{n} \rangle -2\langle z_{n}, Jx_{n} \rangle\leq-2\lambda_{n}\bigl\langle z_{n}-x^{*},Sx_{n}-Sz_{n} \bigr\rangle , \\& 2\bigl\langle x^{*},Jx_{n}-Jz_{n} \bigr\rangle +2 \|z_{n}\|^{2}-2\langle z_{n}, Jx_{n} \rangle\leq-2\lambda_{n}\bigl\langle z_{n}-x^{*},Sx_{n}-Sz_{n} \bigr\rangle , \\& \begin{aligned} \big\| x^{*}\big\| ^{2}-2\bigl\langle x^{*},Jz_{n} \bigr\rangle + \|z_{n}\|^{2}\leq{}& \big\| x^{*} \big\| ^{2}-2\bigl\langle x^{*},Jx_{n} \bigr\rangle + \|x_{n}\|^{2}-\|z_{n}\| ^{2}+2\langle z_{n},Jx_{n} \rangle \\ &-\|x_{n}\|^{2}-2\lambda_{n}\bigl\langle z_{n}-x^{*},Sx_{n}-Sz_{n} \bigr\rangle ,\end{aligned} \\& \phi\bigl(x^{*},z_{n}\bigr)\leq\phi\bigl(x^{*},x_{n} \bigr)-\phi(z_{n},x_{n})-2\lambda _{n}\bigl\langle z_{n}-x^{*},Sx_{n}-Sz_{n} \bigr\rangle . \end{aligned}$$

Since $u_{n}=\Upsilon_{t_{n}}y_{n}$ for all $n\in N\cup\{0\}$ and $\Upsilon_{t_{n}}$ is relatively nonexpansive, we have

3.2 $$\begin{aligned}[b] \phi\bigl(x^{*},u_{n}\bigr)&=\phi\bigl(x^{*}, \Upsilon_{t_{n}}y_{n}\bigr) \\ &\leq\phi\bigl(x^{*},y_{n}\bigr).\end{aligned} $$

Now, we estimate

3.3 $$\begin{aligned}[b] \phi\bigl(x^{*},y_{n}\bigr)={}&\phi\bigl(x^{*},J^{-1} \bigl(\theta_{n}Jx_{n}+(1-\theta _{n})JTz_{n} \bigr)\bigr) \\ ={}&\big\| x^{*}\big\| ^{2}-2\bigl\langle x^{*}, \theta_{n}Jx_{n}+(1-\theta _{n})JTz_{n} \bigr\rangle +\big\| \theta_{n}Jx_{n}+(1-\theta_{n})JTz_{n} \big\| ^{2} \\ \leq{}& \big\| x^{*}\big\| ^{2}-2\theta_{n}\bigl\langle x^{*},Jx_{n}\bigr\rangle -2(1-\theta _{n})\bigl\langle x^{*},JTz_{n}\bigr\rangle +\theta_{n}\|x_{n}\|^{2}+(1- \theta_{n})\|Tz_{n}\|^{2} \\ \leq{}&\theta_{n}\phi\bigl(x^{*},x_{n}\bigr)+(1- \theta_{n})\phi\bigl(x^{*},Tz_{n}\bigr) \\ \leq{}&\theta_{n}\phi\bigl(x^{*},x_{n}\bigr)+(1- \theta_{n})\phi\bigl(x^{*},Tz_{n}\bigr) \\ \leq{}&\theta_{n}\phi\bigl(x^{*},x_{n}\bigr)+(1- \theta_{n})\phi\bigl(x^{*},z_{n}\bigr).\end{aligned} $$

By (3.1), (3.2), and (3.3) we observe that

$$\phi\bigl(x^{*},u_{n}\bigr)\leq\phi\bigl(x^{*},x_{n} \bigr)-(1-\theta_{n})\phi (z_{n},x_{n})-2(1- \theta_{n})\lambda_{n}\bigl\langle z_{n}-x^{*}, Sx_{n}-Sz_{n} \bigr\rangle . $$

This implies that $x^{*}\in P_{n}$. Therefore, $\Gamma\subset P_{n}$ for all $n\in N\cup\{0\}$.

Next, we show by induction that $\Gamma\subset P_{n}\cap Q_{n}$ for all $n\in N\cup\{0\}$. Since $Q_{0}=K$, we have $\Gamma\subset P_{0}\cap Q_{0}$. Suppose that $\Gamma\subset P_{k}\cap Q_{k}$ for some $k\in N\cup\{0\}$. Then there exists $x_{k+1}\in P_{k}\cap Q_{k}$ such that $x_{k+1}=\prod_{P_{k}\cap Q_{k}}x $. From the definition of $x_{k+1}$ we have, for all $z\in P_{k}\cap Q_{k}$,

$$\langle x_{k+1}-z,Jx-Jx_{k+1} \rangle\geq0. $$

Since $\Gamma\subset P_{k}\cap Q_{k}$, we have

$$\langle x_{k+1}-z,Jx-Jx_{k+1} \rangle\geq0 \quad\text{for all } z \in \Gamma, $$

and hence $z\in Q_{k+1}$. So, we have $\Gamma\subset Q_{k+1}$. Therefore, we have $\Gamma\subset P_{k+1}\cap Q_{k+1}$.

Thus, we have that $\Gamma\subset P_{n}\cap Q_{n}$ for all $n\in N\cup \{0\}$. This means that $\{x_{n}\}$ is well-defined.

Further, we show that the sequence $\{x_{n}\}$ converges strongly to $x^{*} =\prod_{\Gamma}x\in\Gamma$.

By the definition of $Q_{n}$ we get $x_{n}=\prod_{Q_{n}}x$. Using $x_{n}=\prod_{Q_{n}}x$ and Lemma 2.1, we have, for all $x^{*}\in \Gamma\subset Q_{n}$,

$$\begin{aligned} \phi(x_{n},x)&=\phi\biggl(\prod_{Q_{n}}x,x \biggr) \\ &\leq\phi\bigl(x^{*},x\bigr)-\phi\biggl(x^{*},\prod _{Q_{n}}x\biggr) \\ &\leq\phi\bigl(x^{*},x\bigr).\end{aligned} $$

Thus $\{\phi(x_{n},x)\}$ is bounded. Therefore $\{x_{n}\}$ is bounded.

Letting $x^{*}\in\Gamma$, we have

$$\begin{aligned} \|Sx_{n}\|&\leq\big\| Sx_{n}-Sx^{*}\big\| + \big\| Sx^{*}\big\| \\ &\leq\frac{1}{\gamma}\big\| x_{n}-x^{*}\big\| +\big\| Sx^{*} \big\| .\end{aligned} $$

So, $\{Sx_{n}\}$ is bounded.

From $\phi(z_{n}, J^{-1}(Jx_{n}-\lambda_{n}Sx_{n}))\leq\phi(x^{*}, J^{-1}(Jx_{n}-\lambda_{n}Sx_{n}))$ we have

$$\begin{aligned} 0&\geq\|z_{n}\|^{2}-2\langle z_{n},Jx_{n}- \lambda_{n}Sx_{n}\rangle-\big\| x^{*} \big\| ^{2}+2\bigl\langle x^{*},Jx_{n}- \lambda_{n}Sx_{n}\bigr\rangle \\ &\geq\|z_{n}\|^{2}-2\|z_{n}\|\bigl( \|x_{n}+\lambda_{n}\|\|Sx_{n}\|\bigr)-\big\| x^{*}\big\| ^{2}-2\big\| x^{*}\big\| \bigl(\|x_{n}\|+ \lambda_{n}\|Sx_{n}\|\bigr).\end{aligned} $$

Denote $M=\sup\{\|x_{n}\|,\|Sx_{n}\|\}$. Now, we have

$$\|z_{n}\|^{2}-M\|z_{n}\|-\big\| x^{*} \big\| ^{2}-M\big\| x^{*}\big\| \leq0 \quad\mbox{for all } n\in N\cup\{0\}. $$

Thus $\{z_{n}\}$ is bounded.

Since $x_{n+1}=\prod_{P_{n}\cap Q_{n}}x\in P_{n}\cap Q_{n}\subset Q_{n}$ and $x_{n}=\prod_{Q_{n}}x$, from the definition of $\prod_{Q_{n}}$ we have

$$\phi(x_{n},x)\leq\phi(x_{n+1},x) \quad\mbox{for all } n\in N\cup \{0\}. $$

Thus $\{\phi(x_{n},x)\}$ is nondecreasing. So, the limit of $\{\phi (x_{n},x)\}$ exists. By the construction of $Q_{n}$ we have $Q_{m}\subset Q_{n}$ and $x_{m}=\prod_{Q_{m}}x\in Q_{n}$ for $m\geq n$. It follows that

3.4 $$\begin{aligned}[b] \phi(x_{m},x_{n})&=\phi\biggl(x_{m},\prod _{Q_{n}}x\biggr) \\ &\leq \phi(x_{m},x)-\phi\biggl(\prod_{Q_{n}}x,x \biggr) \\ &=\phi(x_{m},x)-\phi(x_{n},x).\end{aligned} $$

Letting $m,n\to\infty$, we have $\phi(x_{m},x_{n})\to0$, and hence, applying Lemma 2.6, we have $\|x_{m}-x_{n}\|\to0$ as $m,n\to\infty$. Thus $\{x_{n}\}$ is a Cauchy sequence. Since *X* is a Banach space and *K* is closed and convex, we can assume that $x_{n}\to{x^{*}}\in K$ as $n\to\infty$. From (3.4) we get

$$\phi(x_{n+1},x_{n})\leq\phi(x_{n+1},x)- \phi(x_{n},x) \quad\mbox{for all } n\in N\cup\{0\}, $$

which implies

3.5 $$ \lim_{n\to\infty}\phi(x_{n+1},x_{n})=0. $$

Using Lemma 2.6, we get

3.6 $$ \lim_{n\to\infty}\|x_{n+1}-x_{n}\|=0. $$

By Lemma 2.3 and $x_{n+1}\in P_{n}$ we estimate

$$\begin{aligned} \phi(x_{n+1},u_{n})\leq{}& \phi(x_{n+1},x_{n})- \phi(z_{n},x_{n})-2\lambda _{n}\langle z_{n}-x_{n+1}, Sx_{n}-Sz_{n}\rangle \\ ={}&\phi(x_{n+1},x_{n})-\phi(z_{n},x_{n})-2 \lambda_{n}\langle z_{n}-x_{n}, Sx_{n}-Sz_{n}\rangle \\ &-2\lambda_{n}\langle x_{n}-x_{n+1}, Sx_{n}-Sz_{n}\rangle \\ \leq{}&\phi(x_{n+1},x_{n})-\phi(z_{n},x_{n})+2 \lambda_{n}\|z_{n}-x_{n}\| \|Sx_{n}-Sz_{n} \| \\ &+2\lambda_{n}\|x_{n}-x_{n+1}\| \|Sx_{n}-Sz_{n}\| \\ \leq{}&\phi(x_{n+1},x_{n})+\biggl(\frac{2\lambda_{n}}{\gamma}-c_{1} \biggr)\| x_{n}-z_{n}\|^{2}+\frac{2\lambda_{n}}{\gamma} \|x_{n}-x_{n+1}\|\| x_{n}-z_{n}\|.\end{aligned} $$

Using (3.5), (3.6), and the inequality $\sup_{n\in N}\lambda_{n}< \frac {c_{1}^{2}\gamma}{2}$, we have

$$\lim_{n\to\infty}\biggl(c_{1}-\frac{2\lambda_{n}}{\gamma}\biggr) \|x_{n}-z_{n}\| ^{2}=\lim_{n\to\infty} \phi(x_{n+1},u_{n})=0, $$

which implies

3.7 $$ \lim_{n\to\infty}\|x_{n}-z_{n}\|= \lim _{n\to\infty}\|x_{n+1}-u_{n}\| =0. $$

Using (3.6) and (3.7), we have

3.8 $$ \lim_{n\to\infty}\|x_{n}-u_{n}\|=0. $$

The uniform continuity of *J* implies that

3.9 $$ \lim_{n\to\infty}\|Jx_{n}-Ju_{n}\|=0. $$

Using the property of *ϕ* and Lemma 2.8, we have, for all $x^{*}\in \Gamma$,

3.10 $$\begin{aligned}[b] \phi\bigl(x^{*},y_{n}\bigr)={}&\phi\bigl(x^{*},J^{-1} \bigl(\theta_{n} Jx_{n}+(1-\theta _{n})JTz_{n} \bigr)\bigr) \\ ={}&\big\| x^{*}\big\| ^{2}-2\bigl\langle x^{*}, \theta_{n} Jx_{n}+(1-\theta_{n})JTz_{n} \bigr\rangle +\big\| \theta_{n} Jx_{n}+(1-\theta_{n})JTz_{n} \big\| ^{2} \\ \leq{}&\big\| x^{*}\big\| ^{2}-2\theta_{n}\bigl\langle x^{*}, Jx_{n} \bigr\rangle -2(1-\theta _{n})\bigl\langle x^{*}, JTz_{n} \bigr\rangle +\theta_{n} \|Jx_{n}\|^{2} \\ &+(1-\theta_{n})\|JTz_{n}\|^{2}- \theta_{n}(1-\theta_{n})g\bigl(\| Jx_{n}-JTz_{n} \|\bigr) \\ ={}&\theta_{n}\phi\bigl(x^{*},x_{n}\bigr)+(1- \theta_{n})\phi\bigl(x^{*},z_{n}\bigr)-\theta _{n}(1-\theta_{n})g\bigl(\|Jx_{n}-JTz_{n} \|\bigr).\end{aligned} $$

Next, we estimate

$$\begin{aligned} \phi\bigl(x^{*},z_{n}\bigr)={}&\phi\Bigl(x^{*}, \prod J^{-1}( Jx_{n}-\lambda _{n}Sx_{n}) \Bigr) \\ \leq{}&\phi\bigl(x^{*}, J^{-1}( Jx_{n}- \lambda_{n}Sx_{n})\bigr) \\ ={}&F\bigl(x^{*},Jx_{n}-\lambda_{n}Sx_{n} \bigr) \\ \leq{}&F\bigl(x^{*},(Jx_{n}-\lambda_{n}Sx_{n})+ \lambda_{n}Sx_{n}\bigr)-2\bigl\langle J^{-1}(Jx_{n}- \lambda_{n}Sx_{n})-p, \lambda_{n}Sx_{n} \bigr\rangle \\ ={}&F\bigl(x^{*},Jx_{n}\bigr)-2\lambda_{n}\bigl\langle J^{-1}(Jx_{n}-\lambda _{n}Sx_{n})-x^{*},Sx_{n} \bigr\rangle \\ ={}&\phi\bigl(x^{*},x_{n}\bigr)-2\lambda_{n}\bigl\langle x_{n}-x^{*},Sx_{n} \bigr\rangle -2 \lambda_{n}\bigl\langle J^{-1}(Jx_{n}- \lambda_{n}Sx_{n})-x_{n},Sx_{n} \bigr\rangle \\ \leq{}&\phi\bigl(x^{*},x_{n}\bigr)-2\lambda_{n} \bigl\langle x_{n}-x^{*},Sx_{n}-Sx^{*} \bigr\rangle -2\lambda_{n}\bigl\langle x_{n}-x^{*},Sx^{*} \bigr\rangle \\ &+2\bigl\langle J^{-1}(Jx_{n}-\lambda_{n}Sx_{n})-x_{n},Sx_{n} \bigr\rangle \\ \leq{}&\phi\bigl(x^{*},x_{n}\bigr)-2\lambda_{n} \gamma\big\| Sx_{n}-Sx^{*}\big\| ^{2}+2\bigl\langle J^{-1}(Jx_{n}-\lambda_{n}Sx_{n})-x_{n},Sx_{n} \bigr\rangle . \end{aligned}$$

From this, using Lemma 2.4 and the inequality $\|Sx\|\leq\|Sx-Sx^{*}\|$ for $x\in K$ and $x^{*}\in\Gamma$, we have

3.11 $$\begin{aligned}[b] \phi\bigl(x^{*},z_{n}\bigr)&\leq \phi\bigl(x^{*},x_{n} \bigr)-2\lambda_{n}\gamma\big\| Sx_{n}-Sx^{*} \big\| ^{2}+\frac{4}{c_{1}^{2}}\lambda_{n}^{2} \big\| Sx_{n}-Sx^{*}\big\| ^{2} \\ &=\phi\bigl(x^{*},x_{n}\bigr)+2\lambda_{n} \biggl(\frac{2}{c_{1}^{2}}\lambda_{n}-\gamma \biggr)\big\| Sx_{n}-Sx^{*} \big\| ^{2}.\end{aligned} $$

From (3.11) and (3.10) we have

3.12 $$\begin{aligned}[b] \phi\bigl(x^{*},y_{n}\bigr)\leq{}&\phi\bigl(x^{*},x_{n} \bigr)+2(1-\theta_{n})\lambda_{n}\biggl(\frac {2}{c_{1}^{2}} \lambda_{n}-\gamma\biggr)\big\| Sx_{n}-Sx^{*} \big\| ^{2}\\&-\theta _{n}(1-\theta_{n})g\bigl( \|Jx_{n}-JTz_{n}\|\bigr).\end{aligned} $$

Using (3.2) in (3.12), we have

3.13 $$\begin{aligned}[b] \phi\bigl(x^{*},u_{n}\bigr)\leq{}&\phi\bigl(x^{*},x_{n} \bigr)+2(1-\theta_{n})\lambda_{n}\biggl(\frac {2}{c_{1}^{2}} \lambda_{n}-\gamma\biggr)\big\| Sx_{n}-Sx^{*} \big\| ^{2}\\&-\theta _{n}(1-\theta_{n})g\bigl( \|Jx_{n}-JTz_{n}\|\bigr).\end{aligned} $$

Since $\lambda_{n}\leq\frac{c_{1}^{2}\gamma}{2}$, we get

3.14 $$ \theta_{n}(1-\theta_{n})g\bigl(\|Jx_{n}-JTz_{n} \|\bigr)\leq\phi\bigl(x^{*},x_{n}\bigr)-\phi \bigl(x^{*},u_{n} \bigr). $$

Now,

$$\begin{aligned} \phi\bigl(x^{*},x_{n}\bigr)-\phi\bigl(x^{*},u_{n} \bigr)&=\|x_{n}\|^{2}-\|u_{n}\| ^{2}-2 \bigl\langle x^{*}, Jx_{n}-Ju_{n} \bigr\rangle \\ &\leq \|x_{n}-u_{n}\|\bigl(\|x_{n}\|+ \|u_{n}\|\bigr)+2\big\| x^{*}\big\| \bigl(\|Jx_{n}-Ju_{n} \|\bigr).\end{aligned} $$

It follows from (3.8) and (3.9) that

3.15 $$ \phi\bigl(x^{*},x_{n}\bigr)-\phi\bigl(x^{*},u_{n} \bigr)\to0 \quad\text{as } n\to\infty. $$

Thus, from (3.14) and (3.15) we have

$$g\bigl(\|Jx_{n}-JTz_{n}\|\bigr)\to0 \quad\text{as } n\to\infty. $$

Using Lemma 2.9, we obtain

$$\|Jx_{n}-JTz_{n}\|\to0 \quad\text{as } n\to\infty. $$

Since $J^{-1}$ is uniformly norm-to-norm continuous, we have

3.16 $$ \|x_{n}-Tz_{n}\|\to0 \quad\text{as } n\to\infty. $$

From (3.13) we have

$$2(1-\theta_{n})\lambda_{n}\biggl(\gamma-\frac{2}{c_{1}^{2}} \lambda_{n}\biggr)\big\| Sx_{n}-Sx^{*}\big\| ^{2} \leq\phi\bigl(x^{*},x_{n}\bigr)-\phi\bigl(x^{*},u_{n} \bigr), $$

which implies that

3.17 $$ \lim_{n\to\infty}\big\| Sx_{n}-Sx^{*}\big\| =0. $$

Using Lemmas 2.1 and 2.10 and (3.4), we estimate

3.18 $$\begin{aligned}[b] \phi(x_{n},z_{n})&=\phi\biggl(x_{n}, \prod _{K}J^{-1}(Jx_{n}-\lambda _{n}Sx_{n})\biggr) \\ &\leq\phi\bigl(x_{n}, J^{-1}(Jx_{n}- \lambda_{n}Sx_{n})\bigr) \\ &=F(x_{n},Jx_{n}-\lambda_{n}Sx_{n}) \\ &\leq F\bigl(x_{n},(Jx_{n}-\lambda_{n}Sx_{n})+ \lambda_{n}Sx_{n}\bigr)-2\bigl\langle J^{-1}(Jx_{n}- \lambda_{n}Sx_{n})-x_{n},\lambda_{n}Sx_{n} \bigr\rangle \\ &=\phi(x_{n},x_{n})+2\bigl\langle J^{-1}(Jx_{n}- \lambda _{n}Sx_{n})-x_{n},-\lambda_{n}Sx_{n} \bigr\rangle \\ &=2\bigl\langle J^{-1}(Jx_{n}-\lambda_{n}Sx_{n})-x_{n},- \lambda_{n}Sx_{n} \bigr\rangle \\ &\leq2\big\| J^{-1}(Jx_{n}-\lambda_{n}Sx_{n})-J^{-1}Jx_{n} \big\| \|\lambda _{n}Sx_{n}\|.\end{aligned} $$

By Lemma 2.4, using the inequality $\|Sx\|\leq\|Sx-Sx^{*}\|$ for $x\in K$, $x^{*}\in\Gamma$, we have

$$\phi(x_{n}, z_{n})\leq\frac{4}{c_{1}^{2}\gamma} \big\| Sx_{n}-Sx^{*}\big\| ^{2}. $$

It follows from (3.17) and Lemma 2.6 that

3.19 $$ \lim_{n\to\infty}\|x_{n}-z_{n}\|=0. $$

Thus $z_{n}\to x^{*}$ as $n\to\infty$.

Since $u_{n}= \Upsilon_{t_{n}}y_{n}$, using Lemma 2.11 and (3.11), we get

$$\begin{aligned} \phi(u_{n}, y_{n})={}&\phi(\Upsilon_{t_{n}}y_{n}, y_{n}) \\ ={}&\phi\bigl(x^{*}, y_{n}\bigr)-\phi\bigl(x^{*}, u_{n}\bigr) \\ \leq{}&\theta_{n}\phi\bigl(x^{*}, x_{n}\bigr)+(1- \theta_{n})\phi\bigl(x^{*}, z_{n}\bigr)-\phi \bigl(x^{*}, u_{n}\bigr) \\ \leq{}&\theta_{n}\phi\bigl(x^{*}, x_{n}\bigr) \\ &+(1- \theta_{n})\biggl[\phi\bigl(x^{*},x_{n}\bigr)+2\lambda_{n}\biggl(\frac{2}{c_{1}^{2}}\lambda_{n}-\gamma \biggr)\big\| Sx_{n}-Sx^{*}\big\| ^{2}\biggr]-\phi \bigl(x^{*}, u_{n}\bigr) \\ \leq{}&\phi\bigl(x^{*}, x_{n}\bigr)+2(1- \theta_{n})\lambda_{n}\biggl(\frac {2}{c_{1}^{2}} \lambda_{n}-\gamma\biggr)\big\| Sx_{n}-Sx^{*} \big\| ^{2}-\phi\bigl(x^{*}, u_{n}\bigr).\end{aligned} $$

From this, using (3.15) and the restrictions on the sequences $\{\theta _{n}\}$ and $\{\lambda_{n}\}$, we get

$$\lim_{n\to\infty}\phi(u_{n}, y_{n})=0. $$

By Lemma 2.6,

$$\lim_{n\to\infty}\|u_{n}- y_{n}\|=0. $$

Using the uniform continuity of *J*, we have

3.20 $$ \lim_{n\to\infty}\|Ju_{n}- Jy_{n}\|=0. $$

From (3.16) and (3.19) we get

$$\|Tz_{n}-z_{n}\|\leq\|Tz_{n}-x_{n} \|+\|z_{n}-x_{n}\|\to0 \quad\text{as } n\to\infty, $$

which implies that $x^{*}\in\operatorname{Fix}(T)$.

Further, we show that $x^{*}\in\text{Sol(VIP(1.3))}$. Since $\{x_{n}\}$ is bounded, there exists a subsequence $\{x_{n_{k}}\}$ of $\{x_{n}\}$ that converges weakly to $x^{*}$. Define the mapping $M\subset X\times X^{*}$ as follows:

$$M(z) =\left \{ \textstyle\begin{array}{l@{\quad}l} S(z)+N_{K}(z) &\mbox{if } z \in K , \\ \emptyset &\mbox{if } z \notin K, \end{array}\displaystyle \right . $$

where $N_{K}(z):=\{w\in X:\langle z-x,w\rangle\geq0, \forall x\in K\}$ is the normal cone to *K* at $z \in K$. By Lemma 2.7, *M* is a maximal monotone operator, and $M^{-1}(0)=\operatorname{VI}(K,S)$. Let $(z,w)\in \operatorname{graph}(M)$. Since $w\in M(z)=S(z)+N_{K}(z)$, we get $w-Sz\in N_{K}(z)$. Since $z_{n}\in K$, we obtain

3.21 $$ \langle z-z_{n_{k}},w-Sz\rangle\geq0. $$

On the other hand, $z_{n_{k}}=\prod_{K}J^{-1}(Jx_{n_{k}}-\lambda _{n_{k}}Sx_{n_{k}})$, and using Lemma 2.1, we obtain

$$\bigl\langle z-z_{n_{k}},Jz_{n_{k}}-(Jx_{n_{k}}- \lambda_{n_{k}}Sx_{n_{k}}) \bigr\rangle \leq0, $$

and thus

3.22 $$ \biggl\langle z-z_{n_{k}},\frac{Jx_{n_{k}}-Jz_{n_{k}}}{\lambda _{n_{k}}}-Sx_{n_{k}} \biggr\rangle \leq0. $$

Therefore, it follows from the monotonicity of *S*, (3.21), and (3.22) that

$$\begin{aligned} \langle z-z_{n_{k}}, w\rangle\geq{}&\langle z-z_{n_{k}},Sz\rangle \\ \geq{}&\langle z-z_{n_{k}},Sz\rangle+\biggl\langle z-z_{n_{k}}, \frac {Jx_{n_{k}}-Jz_{n_{k}}}{\lambda_{n_{k}}}-Sx_{n_{k}} \biggr\rangle \\ ={}&\biggl\langle z-z_{n_{k}},Sz-Sx_{n_{k}}+\frac {Jx_{n_{k}}-Jz_{n_{k}}}{\lambda_{n_{k}}} \biggr\rangle \\ ={}&\langle z-z_{n_{k}},Sz-Sz_{n_{k}}\rangle+\langle z-z_{n_{k}},Sz_{n_{k}}-Sx_{n_{k}}\rangle +\biggl\langle z-z_{n_{k}},\frac{Jx_{n_{k}}-Jz_{n_{k}}}{\lambda _{n_{k}}}\biggr\rangle \\ \geq{}&{-}\|z-z_{n_{k}}\|\|Sz_{n_{k}}-Sx_{n_{k}}\|- \|z-z_{n_{k}}\|\bigg\| \frac {Jx_{n_{k}}-Jz_{n_{k}}}{a}\bigg\| \\ \geq{}&{-}\frac{1}{\gamma}\|z-z_{n_{k}}\|\|z_{n_{k}}-x_{n_{k}} \|-\| z-z_{n_{k}}\|\frac{\|Jx_{n_{k}}-Jz_{n_{k}}\|}{a} \\ \geq{}&{-}\rho\biggl(\frac{1}{\gamma}\|z_{n_{k}}-x_{n_{k}}\|+ \frac{\| Jx_{n_{k}}-Jz_{n_{k}}\|}{a}\biggr), \end{aligned}$$

where $\rho=\sup_{k\in N}\{\|z-z_{n_{k}}\|\}$ and $a<\limsup\lambda _{n}$. Taking the limit as $k\to\infty$ and using the fact that $\{\| z-z_{n_{k}}\|\}_{k\in N}$ is bounded, we see that $\langle z-x^{*},w \rangle\geq0 $. Thus $x^{*}\in\text{Sol(VIP(1.3))}$.

Next, we prove that $x^{*}\in\text{Sol(GEP(1.1))}$.

The relation $u_{n}=\Upsilon_{t_{n}}y_{n}$ implies that

$$G(u_{n},y)+\xi(y,u_{n})-\xi(u_{n},u_{n})+ \frac{1}{t_{n}}\langle y-u_{n}, Ju_{n}-Jy_{n} \rangle\geq0 \quad\mbox{for all } y\in K. $$

Let $y_{p}=(1-p)x^{*}+py$ for $p\in(0,1]$. Since $y\in K$ and $x^{*}\in K$, we get $y_{p}\in K$, and hence

$$G(u_{n},y_{p})+\xi(y_{p},u_{n})- \xi(u_{n},u_{n})+\frac {1}{t_{n}}\langle y_{p}-u_{n}, Ju_{n}-Jy_{n}\rangle \geq0. $$

Using (3.20) and $\liminf_{n\to\infty}t_{n}>0 $, we have

$$\lim_{n\to\infty}\frac{\|Jy_{n}-J\Upsilon_{t_{n}}y_{n}\|}{t_{n}}=0. $$

Further, since *ξ* is weakly continuous and *G* is weakly lower semicontinuous in the second argument, letting ${n\to\infty}$, we get

$$\begin{gathered} G\bigl(x^{*},y_{p}\bigr)+\xi\bigl(y_{p},x^{*} \bigr)-\xi\bigl(x^{*},x^{*}\bigr) \geq 0, \\ \xi\bigl(y_{p},x^{*}\bigr)-\xi\bigl(x^{*},x^{*} \bigr)\geq G\bigl(y_{p},x^{*}\bigr).\end{gathered} $$

Now, for $p > 0$,

$$\begin{aligned} 0&=G(y_{p}, y_{p}) \\ &\leq p G(y_{p}, y)+(1-p)G\bigl(y_{p}, x^{*} \bigr) \\ &\leq p G(y_{p}, y)+(1-p)\bigl[\xi\bigl(y_{p},x^{*} \bigr)-\xi\bigl(x^{*},x^{*}\bigr)\bigr] \\ &\leq p G(y_{p}, y)+(1-p)p\bigl[\xi\bigl(y,x^{*}\bigr)-\xi \bigl(x^{*},x^{*}\bigr)\bigr] \\ &\leq p \bigl[G(y_{p}, y)+(1-p) \bigl(\xi\bigl(y,x^{*} \bigr)-\xi\bigl(x^{*},x^{*}\bigr)\bigr)\bigr].\end{aligned} $$

Dividing by $p>0$ and letting $p\to0_{+}$, we have

$$G\bigl(x^{*},y\bigr)+\xi\bigl(y,x^{*}\bigr)-\xi \bigl(x^{*},x^{*}\bigr)\geq0. $$

Thus, $x^{*}\in\text{Sol(GEP(1.1))}$, and hence $x^{*}\in\Gamma$. □

Finally, we have the following consequences of Theorem 3.1.

### Corollary 3.1

*Let* *X* *be a* 2-*uniformly convex and uniformly smooth Banach space*, *and let* *K* *be a nonempty closed and convex subset of* *X*. *Let* $S:K\to X^{*}$ *be a* *γ*-*inverse strongly monotone mapping with constant* $\gamma\in(0,1)$, *let* $G:K\times K\to \mathbf{R}$ *be a nonlinear mapping satisfying Assumption* 2.1(i)-(iv), *and let* $T:K\to K$ *be a relatively nonexpansive mapping such that* $\Gamma:=\textit{Sol}(\textit{EP}(\text{1.2}))\cap\textit{Sol}(\textit{VIP}(\text{1.3}))\cap \operatorname{Fix}(T) \neq\emptyset$. *Let the iterative sequence* $\{ x_{n}\}$ *be generated as follows*:

$$\begin{gathered} x_{0}=x\in K , \\ z_{n}=\prod_{K}J^{-1}(Jx_{n}- \lambda_{n}Sx_{n}), \\ y_{n}=J^{-1}\bigl(\theta_{n}Jx_{n}+(1- \theta_{n})JTz_{n}\bigr), \\ u_{n}\in K \end{gathered}$$

*such that*

$$\begin{gathered}G(u_{n},y)+ \frac{1}{t_{n}}\langle y-u_{n},Ju_{n}-Jy_{n} \rangle\geq0,\quad \forall y\in K, \\ P_{n}=\bigl\{ z\in K:\phi(z,u_{n})\leq \phi(z,x_{n})-(1-\theta_{n})\phi (z_{n},x_{n}) -2(1-\theta_{n})\lambda_{n}\langle z_{n}-z, Sx_{n}-Sz_{n}\rangle\bigr\} , \\ Q_{n}=\bigl\{ z\in K:\langle x_{n}-z,Jx-Jx_{n} \rangle\geq0\bigr\} , \\ x_{n+1}=\prod_{P_{n}\cap Q_{n}}x, \quad\forall n\in N\cup \{0\},\end{gathered} $$

*where* *J* *is the normalized duality mapping on* *X*, $t_{n}\in(0,\infty )$, *and* $\{\lambda_{n}\}$ *and* $\{\theta_{n}\}$ *are the sequences in* $(0, \infty)$ *and* (0,1) *satisfying the following*:

(i) $0<\liminf_{n\to\infty}\lambda_{n}\leq\limsup_{n\to\infty }\lambda_{n}<\frac{c_{1}^{2}\gamma}{2}$, *where* $c_{1}$ *is the constant in Lemma* 2.2;
(ii) $0<\liminf_{n\to\infty}\theta_{n}\leq\limsup_{n\to\infty }\theta_{n}<1 $.

*Then*, $\{x_{n}\}$ *converges strongly to* $\prod_{\Gamma} x$.

### Proof

The proof follows by taking $\xi=0$ in Theorem 3.1. □

### Corollary 3.2

*Let* *X* *be a* 2-*uniformly convex and uniformly smooth Banach space*, *and let* *K* *be a nonempty closed and convex subset of* *X*. *Let* $S:K\to X^{*}$ *be a* *γ*-*inverse strongly monotone mapping with constant* $\gamma\in(0,1)$, *and let* $T:K\to K$ *be a relatively nonexpansive mapping such that* $\Gamma:= \textit {Sol}(\textit{VIP}(\text{1.3}))\cap\operatorname{Fix}(T) \neq\emptyset$. *Let the iterative sequence* $\{x_{n}\}$ *be generated as follows*:

$$\begin{aligned}& x_{0}=x\in K, \\& z_{n}=\prod_{K}J^{-1}(Jx_{n}- \lambda_{n}Sx_{n}), \\& y_{n}=J^{-1}\bigl(\theta_{n}Jx_{n}+(1- \theta_{n})JTz_{n}\bigr), \\& P_{n}=\bigl\{ z\in K:\phi(z,u_{n})\leq \phi(z,x_{n})-(1-\theta_{n})\phi (z_{n},x_{n}) -2(1-\theta_{n})\lambda_{n}\langle z_{n}-z, Sx_{n}-Sz_{n}\rangle\bigr\} , \\& Q_{n}=\bigl\{ z\in K:\langle x_{n}-z,Jx-Jx_{n} \rangle\geq0\bigr\} , \\& x_{n+1}=\prod_{P_{n}\cap Q_{n}}x,\quad \forall n\in N\cup \{0\}, \end{aligned}$$

*where* *J* *is the normalized duality mapping on* *X*, *and* $\{\lambda_{n}\} $ *and* $\{\theta_{n}\}$ *are sequences in* $(0, \infty)$ *and* $(0,1)$ *satisfying the following*:

(i) $0<\liminf_{n\to\infty}\lambda_{n}\leq\limsup_{n\to\infty }\lambda_{n}<\frac{c_{1}^{2}\gamma}{2}$, *where* $c_{1}$ *is the constant in Lemma* 2.2;
(ii) $0<\liminf_{n\to\infty}\theta_{n}\leq\limsup_{n\to\infty }\theta_{n}<1 $.

*Then*, $\{x_{n}\}$ *converges strongly to* $\prod_{\Gamma} x$.

### Proof

The proof follows by taking $\xi=0$ and $G=0$ in Theorem 3.1. □

## Conclusion

In this paper, we propose an iterative algorithm to find the common solution of the generalized equilibrium problem, variational inequality problem, and fixed point problem for a relatively nonexpansive mapping in a real Banach space. Further, using the hybrid projection method, we proved the strong convergence of the sequences generated by the iterative algorithm. Finally, we derived some consequences from our main result. The result presented in this paper is an improvement and extension of the corresponding results of [3, 4, 8–10].

## References

[1] Takahashi W, Zembayashi K (2009). Strong and weak convergence theorems for equilibrium problems and relatively nonexpansive mappings in Banach spaces. Nonlinear Anal..

[2] Petrot N, Wattanawitoon K, Kumam P (2010). A hybrid projection method for generalized mixed equilibrium problems and fixed point problems in Banach spaces. Nonlinear Anal..

[3] Takahashi S, Takahashi W (2007). Viscosity approximation method for equilibrium problems and fixed point problems in Hilbert space. J. Math. Anal. Appl..

[4] Djafari Rouhani B, Farid M, Kazmi KR (2016). Common solution to generalized mixed quilibrium problem and fixed point problem for a nonexpansive semigroup in Hilbert space. J. Korean Math. Soc..

[5] Blum E, Oettli W (1994). From optimization and variational inequalities to equilibrium problems. Math. Stud..

[6] Nadezhkina N, Takahashi W (2006). Strong convergence theorem by a hybrid method for nonexpansive mappings and Lipschitz-continuous monotone mappings. SIAM J. Optim..

[7] Nakajo K (2015). Strong convergence for gradient projection method and relatively nonexpansive mappings in Banach spaces. Appl. Math. Comput..

[8] Kazmi KR, Rizvi SH (2012). A hybrid extragradient method for approximating the common solutions of a variational inequality, a system of variational inequalities, a mixed equilibrium problem and a fixed point problem. Appl. Math. Comput..

[9] Tada A, Takahashi W, Takahashi W, Tanaka T (2006). Strong convergence theorem for an equilibrium problem and a nonexpansive mapping. Nonlinear Analysis and Convex Analysis.

[10] Matsushita S, Takahashi W (2004). Weak and strong convergence theorems for relatively nonexpansive mappings in Banach spaces. Fixed Point Theory Appl..

[11] Xu HK (1991). Inequalities in Banach spaces with applications. Nonlinear Anal..

[12] Alber YI, Kartosatos AG (1996). Metric and generalized projection operators in Banach spaces. Theory and Applications of Nonlinear Operators of Accretive and Monotone Type.

[13] Kamimura S, Takahashi W (2002). Strong convergence of a proximal-type algorithm in a Banach space. SIAM J. Optim..

[14] Rockafellar RT (1976). Monotone operators and the proximal point algorithm. SIAM J. Control Optim..

[15] Zálinescu C (1983). On uniformly convex functions. J. Math. Anal. Appl..

